# Clinical practice guidelines for neonatal therapeutic hypothermia across European Society for Paediatric and Neonatal Intensive Care (ESPNIC) member countries: a multidisciplinary blind comparison

**DOI:** 10.1016/j.lanepe.2026.101874

**Published:** 2026-09-22

**Authors:** Daniele De Luca, Costanza Neri, Almudena Alonso-Ojembarrena, Mariachiara Ippolito, Anna Milan, Marco Piastra, Manuel Sanchez-Luna, Karl F. Schettler, Anna Zanin, Tullio Ghi, Andrea Cortegiani, Neonatal Critical Care Section of the European Society for Pediatric and Neonatal Intensive Care

**Affiliations:** a Division of Paediatrics and Neonatal Critical Care, “A. Béclère” Medical Centre, Paris Saclay University Hospital, APHP, Paris, France; b Physiopathology and Therapeutic Innovation Unit, INSERM UMR-S1358, Paris Saclay University, Paris, France; c School of Paediatrics, University of Chieti-Pescara “G.D'Annunzio”, Italy; d Neonatal Intensive Care Unit, Puerta del Mar University Hospital, Cádiz, Spain; e Biomedical Research and Innovation Institute of Cádiz (INiBICA), Research Unit, Puerta del Mar University Hospital, Cádiz, Spain; f Department of Precision Medicine in Medical, Surgical and Critical Care (Me.Pre.C.C), University of Palermo, Palermo, Italy; g Department of Anaesthesia, Intensive Care and Emergency, Policlinico Paolo Giaccone, Palermo, Italy; h Neonatal Unit, Evelina London Children Hospital-Guy's & St.Thomas' Hospital, London, UK; i Department of Biotechnology, Critical and Perioperative Medicine, Catholic University of the Sacred Heart, Rome, Italy; j Division of Neonatology, “Gregorio Marañon” University Hospital, Universidad Complutense, Madrid, Spain; k Children Hospital St. Marien, LA-Regio-Kliniken Landshut, Teaching Affiliate of the Ludwig-Maximilians-University of Munich, Munich, Germany; l Department of Women, Children and Adolescent, Hôpitaux Universitaires Genève and University of Geneve, Geneve, Switzerland; m Department of Woman and Infant Sciences Health, Catholic University of Sacred Heart, Rome, Italy

**Keywords:** Perinatal asphyxia, Newborn, Encephalopathy, Neuroprotection

## Abstract

**Background:**

Therapeutic hypothermia (TH) decreases mortality and neuro-developmental disability in neonates greater or equal than 36 weeks’ gestation with moderate-to-severe hypoxic-ischaemic encephalopathy (HIE) due to perinatal asphyxia. TH is often applied in different ways, as national guidelines and target populations may significantly differ. We aimed to compare TH guidelines from various European countries as we hypothesised that guidelines are significantly different and often of insufficient quality.

**Methods:**

Multidisciplinary, blind, critical appraisal of clinical practice guidelines using AGREE-II and RIGHT tools to assess their quality and completeness of the reporting, respectively. The appraisal was performed by neonatologists, obstetricians, paediatric and adult intensivists. The raters were kept unaware of the identity of the other panellists and each of them performed the evaluation of the guidelines independently.

**Findings:**

Total AGREE-II score was different amongst the guidelines (*p* = 0.008): the German guidelines scored highest (5.2 (95% CI: 4.7–5.7), while the others had comparable intermediate scores (approximately between 2.2 and 4.4). A good score in the rigour of development was not achieved by any guideline (German guidelines scored “moderate”). The overall completeness of reporting was different amongst the guidelines, with total completeness ranging between 20% and 49% (*p* < 0.0001). Completeness of reporting was incomplete for most rater-item responses. German and French guidelines had the best domain-based reporting completeness for AGREE-II and RIGHT, respectively. Inter-rater agreement was satisfactory (intraclass correlation coefficient varying between 0.83 and 0.91; *p* < 0.001).

**Interpretation:**

European guidelines for neonatal TH have a general insufficient quality and completeness of reporting.

**Fundings:**

None.

**Research in context**

**Evidence before this study**

Therapeutic hypothermia (TH) decreases mortality and neuro-developmental disability in neonates greater or equal than 36 weeks’ gestation with moderate-to-severe hypoxic-ischaemic encephalopathy (HIE) due to perinatal asphyxia. Nonetheless, in the daily practice, TH is being applied in different ways, as target populations and national guidelines often differ (for instance, for the entry criteria as well as for patient monitoring and management). This variability can have relevant epidemiological, pharmaco-economic, public health and medico-legal implications and deserves to be investigated. Guidelines issued by national authorities or scientific societies from the countries that constitute the largest contribution to ESPNIC membership were analysed. To increase representativeness, we additionally performed a survey of ESPNIC members participating to our annual congress (June 16–18, 2026). We anticipated that most of the clinical practice guidelines were not published in peer-reviewed journals and were impossible to find with a classical literature search. Nonetheless, we also searched PubMed (on June 23, 2026) to verify if any guideline was fully published as an academic article. The search used the following MeSH terms and/or key words: “therapeutic hypothermia” and/or “cooling”, “neonate” and/or “newborn”, “guidelines” and/or “recommendations” and/or “review” and the country name, without year or language limitation. We also searched the references cited in the reviews retrieved by this search.

**Added value of this study**

We found that European guidelines for neonatal TH have a generally insufficient quality and completeness of reporting. The German guidelines scored best for quality but a good score in the rigour of development was not achieved by any guideline. No guideline showed satisfactory global completeness of reporting. However, the French guidelines scored the highest domain-based completeness. Applying these guidelines without a critical appraisal might lead to under- and over-treatment or induce potentially unsafe decisions in special cases (e.g. late preterm patients or neonates beyond the first 6 h of life) and about the general critical care management of these patients. Therefore, no guideline can be recommended or directly applied without critical appraisal and modifications.

**Implications of all the available evidence**

These results are useful for clinicians, researchers, and policymakers. They can be used by national or continental organisations to harmonise practices in Europe and create guidelines more adherent to available evidence and known pathophysiology, using strict criteria that are already used in adult critical care medicine. They help focussing on higher-quality evidence and designing studies to investigate areas where the available knowledge is still insufficient. These findings are also helpful for clinicians at the bedside to critically appraise the guidelines and make more informed choices in some borderline cases.

## Introduction

Therapeutic hypothermia (TH) decreases mortality and neuro-developmental disability in neonates greater than or equal to 36 weeks’ gestation with moderate-to-severe hypoxic-ischaemic encephalopathy (HIE) due to perinatal asphyxia.^1^ As such, TH represents one of the few life-saving and quality-of-life-improving innovations in neonatal intensive care units (NICU) and has become a standard of care.^2^

Nonetheless, in the daily NICU life, TH is being applied in different ways. For instance, the eligibility criteria vary between European countries as reflected in differences amongst national guidelines.^3^ Some of these differences are due to the inconsistencies in the entry criteria of the original TH trials, but also to local customs leading to different interpretations. For example, amplitude-integrated EEG (aEEG) findings and vital parameters are given different weights and their interpretation mainly relies on clinicians’ expertise.^3^ Differences also exist about the general NICU management of cooled neonates. Furthermore, the target population is heterogeneous, ranging from patients with life-threatening multi-organ failure to those with isolated moderate HIE without other organ involvement which makes it difficult to develop detailed, scientifically based guidelines.

This variability means that patients can receive different care depending on where they are born. This may also have relevant epidemiological, pharmaco-economic, public health and medico-legal implications.^4^ TH is an effective and lifesaving intervention, but it is also highly technological and demanding. Therefore, it should be applied in accordance with high-quality clinical practice guidelines, which should be based on strong evidence wherever possible. At the very least, under- and over-treatment must be avoided, and the management should be tailored to the patient's pathophysiology.

To promote a wise use of TH based on scientifically robust and evidence-based criteria, the Neonatal Critical Care Section of the European Society for Paediatric and Neonatal Intensive Care (ESPNIC) designed a project to compare clinical practice guidelines from various European countries, highlighting their differences and areas for improvement. We hypothesised that guidelines were significantly different and often of insufficient quality.

## Methods

### General design

This was a multidisciplinary critical appraisal of TH clinical practice guidelines from the European countries that are represented within ESPNIC membership. The study was based on a convenience sample of European guidelines and does not represent the majority of European countries although these countries would correspond to a significant amount of the continent population. The quality of the guidelines was assessed using the AGREE-II instrument and the completeness of the reporting was evaluated according to the RIGHT statement.^5^^,^^6^ We used both AGREE-II and RIGHT tools to provide a comprehensive evaluation of guidelines, as recommended by specific literature on this topic.^7^ The project was endorsed following relevant internal ESPNIC procedures. The project was carried out using communication tools supported by ESPNIC, with no involvement from any industry or sponsor.

### Panel and guidelines selection

The work was coordinated by two senior researchers (a neonatologist and a methodologist). A multidisciplinary panel of raters was created: the panellists were required to have at least 10 years' experience working in academic NICUs referral for TH with the exception of one member who was permitted to be a trainee representative (i.e. a resident or junior fellow with less than two years' post-residency experience). Furthermore, given the multidisciplinary nature of the topic, the panel was expanded with the following additional panellists: one paediatric intensivist, since NICU and paediatric intensive care units (PICU) are often merged in an unique ward in some European countries,^8^ and neonates represent a significant proportion of PICU admissions^9^; one obstetrician, given that TH treats a condition of perinatal origin and one adult intensivist, since the experience in the use of TH is wider in critical care medicine.^10^ The inclusion of these three panellists was in line with the recent recommendations of the Lancet Child Health Commission for the Future of neonatology who advocated for more cross-disciplinarity especially with obstetrics and foetal medicine as well as with adult and paediatric critical care.^2^ As per ESPNIC policy, gender ratio and geographical distribution were fairly respected and the neonatologists in the panel were all ESPNIC members.

Finally, we made sure that none of the panellists were involved in the original work leading to the preparation and issuance of the evaluated guidelines. Thus, this variegated composition was chosen to be as impartial as possible, representing the pragmatic blend of expertise and competence encountered in clinical practice during the discussions about TH. Details about panellists are provided in Supplementary Table S1. The Chair of ESPNIC Neonatal Critical Care Section (MSL) was not a rater and did not vote for the guidelines evaluation, but was eligible for authorship, having contributed to the conceptualisation of the project, communication within the panel, collection and analysis of data, interpretation of the results, general supervision and critical review of the manuscript.

### Choice of guidelines and representativeness

The more recent version of TH guideline of the countries that constitute the largest contribution to ESPNIC membership (i.e. United Kingdom, Italy, Spain, Switzerland, Germany, Netherlands and France) was originally obtained in its original language, from the national scientific societies or relevant authorities. To increase representativeness, we additionally performed a survey of ESPNIC members participating to our annual congress (from June 16 to June 18, 2026). The survey was conducted during the congress to increase its diffusion (response rate was ≈75%) and revealed that amongst the other countries represented in ESPNIC, Norway, Portugal, Slovakia and Turkey also had guidelines issued by national scientific societies or authorities. Thus, the guidelines of these four countries were added to the comparison.

We anticipated that most of the clinical practice guidelines were not published in peer-reviewed journals and were impossible to find with a classical literature search. Nonetheless, we then also performed a targeted literature search to verify if any of the chosen guideline was fully published as an academic article in a peer-reviewed journal. The search was performed on June 23, 2026, on PubMed using the following MeSH terms and/or key words: “therapeutic hypothermia” and/or “cooling”, “neonate” and/or “newborn”, “guidelines” and/or “recommendations” and/or “review” and the country name, without year or language limitation. We also searched the references cited in the reviews retrieved by this search.

### Critical appraisal

Two authors (DDL and CN) independently extracted the main guidelines characteristics and cross-verified them: these characteristics are summarised into the Supplementary Table S2 (criteria to start TH), Supplementary Table S3 (special cases for “off label” use of TH) and Supplementary Table S4 (characteristics of general NICU management during TH). A dedicated online google drive was created to provide these tables and the full original guidelines to each panellist.^11^ Access to an online artificial intelligence-based tool was also provided to translate the guidelines into English and ease their evaluation.

All panellists conducted an independent assessment of each guideline following AGREE-II and RIGHT methodologies. AGREE-II tool consists of 23 items grouped in 6 domains (purpose of the guideline, stakeholder involvement, rigour of development, clarity of presentation, applicability and editorial independence) as detailed elsewhere,^5^ and scored using a 7-point Likert scale and a comment section. In addition, an overall assessment of the quality was provided, informed primarily by the pattern of domain scores—particularly rigour of development—and was not derived from a numerical threshold.^12^ The RIGHT tool evaluates the completeness and transparency of guideline reporting on 37 items grouped in 7 domains (basic information, background of the guideline, appraised evidence, presentation of the recommendations, external review and quality assurance, funding and conflict of interest, other information) evaluated as “complete”, “incomplete” or “unclear”.^6^

Panellists were kept blinded to the identity of every other panellist until the results of the evaluations were shared. The co-chairs trained all panellists about the methodology (the training lasted 1 h on average). Individual discussions were held between each panellist and a chair before the evaluations to avoid misunderstandings and the co-chairs remained available during the entire process to solve any doubt and retrain the raters, if needed. The methodologist supervised the process but did not vote. As there were nine voting members on the panel, the likelihood of inter-rater variability directly affecting the results was reduced.^13^ In fact, this larger panel, besides ensuring multidisciplinary representativeness, increased the robustness of the appraisal, as aggregation across a larger number of independent raters reduces the influence of individual rater-specific variability on the resulting domain scores, as suggested by a previous study with a similar panel size.^14^ All appraisals were performed electronically and anonymously (i.e. each panellist was not informed of anyone else's evaluation).

### Data analysis and statistics

Results of the AGREE-II evaluations were reported as the median total score and the standardised score for each domain, as per the AGREE-II user manual.^12^ As there is no universally accepted cut-off for the AGREE-II standardised domain score, we have chosen to consider a domain as high, moderate or low quality if it scores >60%, between 40% and 60%, or <40%, respectively.^15^ Regarding the RIGHT evaluations, we descriptively summarised the distribution of all rater-item responses for transparency of the overall evaluation. We then performed a domain-level analyses using the “majority rule” as previously reported.^6^^,^^16^ In other words, in this domain-based analysis, the items were considered successfully reported if a majority of raters rated them as ‘complete’. For visualisation purposes, the domain reporting completeness was displayed using a traffic-light heat map with the same *a priori* thresholds used for AGREE-II results: >60% (green), 40–60% (yellow), <40% (red).^15^ These thresholds were selected pragmatically to aid the interpretation, consistent with common practice of using 60% as a benchmark for higher reporting completeness in checklist-based reporting evaluations.

Descriptive statistics were used to summarise the characteristics of the guidelines and the results of the appraisals: medians (25th–75th percentiles) and frequencies (%), were used for continuous and categorical variables, respectively. AGREE-II data were analysed with Friedman test (which is the non-parametric equivalent of repeated measures-ANOVA, hence more appropriate for non-normally distributed data such as the total AGREE-II score), followed by a Conover–Iman *post hoc* test if appropriate (i.e. if the overall Friedman test is significant), with Holm correction applied to control the family wise error rate in multiple comparisons. RIGHT data were compared using Chi-squared (χ^2^) test of independence on the pooled rater-item responses.

Inter-rater reliability was assessed using a two-way random-effects intraclass correlation coefficient (ICC) for absolute agreement with 95% confidence intervals (CI). Calculations and statistical analyses were performed with Microsoft Excel for Mac 16.101.3 (Redmond–WA, USA) and JASP 0.17.1 (JASP Team (2023), Amsterdam, The Netherlands), respectively. *p* < 0.05 were considered statistically significant.

## Results

Table 1 reports the basic characteristics of the 11 evaluated guidelines. Several presented unclear characteristics or lacked basic information. The French and Italian guidelines were also published as full articles.^17^^,^^18^ A position paper providing some details and advices to foster the Spanish guidelines implementation was also published in a peer-reviewed journal.^19^ No other articles were identified by the literature search. Supplementary Tables S2, S3 and S4 respectively summarise the criteria to start TH, the use of TH in special cases (“off label”, such as patients admitted after the first 6 h of life or late preterm) and the general NICU management during TH. These tables show several incongruencies amongst the guidelines. For instance, the reviewed guidelines for initiating hypothermia: 1) dictate different thresholds for base excess, pH and lactate, or even recommend not trusting cord blood gas; and 2) consider the neurological examination in different ways, giving variable importance to the items in Sarnat's score (or using a different score), or to amplitude-integrated EEG findings. These differences are further accentuated in special cases (e.g. late (>6 h of life) hypothermia and late preterm neonates) and in relation to general critical care management. No guideline provided any guidance regarding the use of TH during extracorporeal life support.

**Table 1:** Basic characteristics of the guidelines.

Country	Year	Peer-reviewed	Consensus methodology	Grading methodology	Year of the last trial cited
France	2010	Yes	Unclear	Not mentioned	2009
Germany	2013	Unclear	Delphi	Not mentioned	2011
Italy	2023	Unclear	Unclear	Unclear	2011
Netherlands	2021	Unclear	Unclear	Not mentioned	2017
Norway	2022	Not mentioned	Not mentioned	Not mentioned	2009
Portugal	2023	Not mentioned	Not mentioned	Not mentioned	2015
Slovakia	2025	Not mentioned	Not mentioned	Not mentioned	2025
Spain	2023	Not mentioned	Not mentioned	Not mentioned	Not mentioned
Switzerland	2011	Not mentioned	Not mentioned	Not mentioned	Not mentioned
Turkey	2018	Not mentioned	Not mentioned	Not mentioned	2014
UK	2020	Not mentioned	Unclear	Unclear	2009

Guidelines are listed per country alphabetic order. ‘Not mentioned’ refers to the complete lack of data about a certain feature; ‘unclear’ means that some information was given without sufficient details or congruence.

Fig. 1 describes results of the quality analysis. There were significant differences in the total AGREE-II score amongst the guidelines (overall *p* = 0.008), however, no pairwise *post hoc* comparisons remained statistically significant after adjustment for multiple testing using the Holm procedure. The German (5.2 (95% CI: 4.7–5.7) and Norwegian (2.57 (95% CI: 2.2–4) guidelines scored highest and lowest, respectively, while the other guidelines had comparable intermediate scores. The standardised score for each domain shows that all guidelines had a low rigour score, except for the German guidelines, which anyway did not reach 50%. Most guidelines received a similar intermediate evaluation, whereas the Swiss guideline had no high-quality domains. Consistent with these results, the German guidelines were attributed an overall medium quality and the highest total score. The inter-rater agreement was fair (ICC = 0.831 (0.775–0.872); *p* < 0.001). Open comments given by all raters are reported in the Supplementary Table S5.

**Fig. 1:**
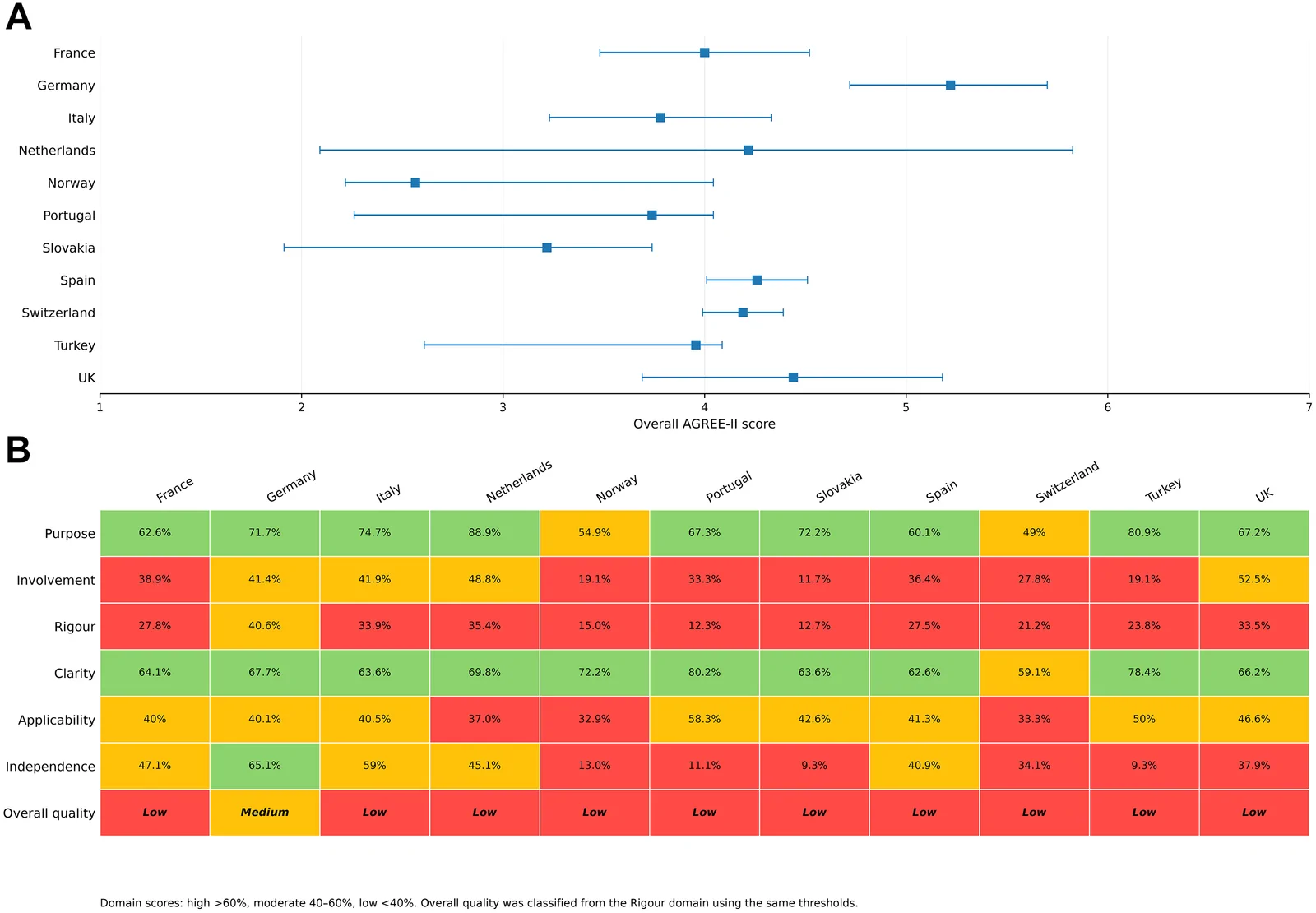
**Quality of the guidelines. Panel A and B represent the total AGREE-II score and the standardised AGREE-II score per each domain, respectively.** Guidelines are listed per country alphabetic order. In **panel A**, the AGREE-II score is calculated as median of all raters and all items. Squares and T-bars represent median values and 95% confidence intervals, respectively. Overall differences among guidelines were significant (Friedman test, *p* = 0.008), but no pairwise *post hoc* comparisons remained significant after Holm correction. In **panel B**, the standardised scores (in %) per each domain are visually reported considering a domain as high (green), moderate (yellow) or low (red) quality if it scores >60%, between 40% and 60%, or <40%, respectively^15^ (more methodological details in the text). All data are expressed as dimensionless variables.

Fig. 2 describes the completeness of reporting. There is a significant difference in the overall completeness of reporting amongst the guidelines with total completeness ranging between 20% and 49% (*p* < 0.001), but for none of them the reporting is considered complete on the majority of rater-item responses. Although this overall summary is purely descriptive, it provides a general overview that is consistent with the following completeness of the domain-specific reporting. At the domain level, completeness also varied widely across the guidelines. French and Swiss guidelines had the highest and lowest domain-based reporting completeness, respectively. The other guidelines received similar intermediate evaluations. Overall, the majority of domains showed low reporting completeness, and seven were classified as ‘unclear’, indicating persistent ambiguity in reporting even when consensus was not reached. The inter-rater agreement was satisfactory (ICC = 0.909 (0.890–0.924); *p* < 0.001).

**Fig. 2:**
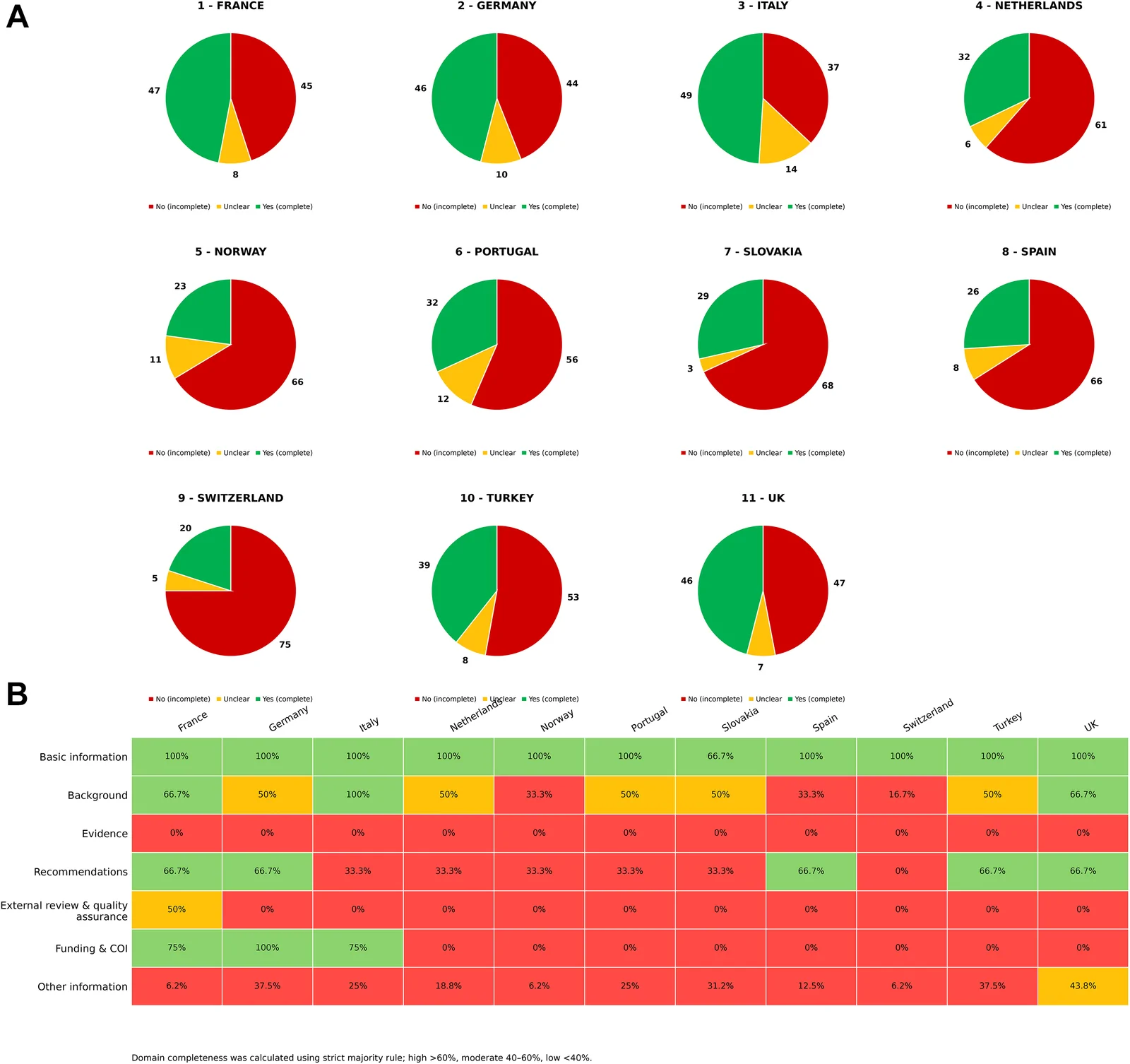
**Completeness of the reporting. Panel A and B illustrate the RIGHT overall and the domain-specific completeness, respectively.** Guidelines are listed per country alphabetic order. In **panel A**, distributions of all rater-item responses to the RIGHT instrument are reported as median percentage of the evaluations given by all raters. Red and green section of the pie charts represent the absence (rater's answer = ‘incomplete’) and the presence (rater's answer = ‘complete’) of the evaluated item, respectively; the yellow section represents an unclear situation (rater's answer = ‘unclear’). Pie charts report results for each national guideline. Evaluations are significantly different between them (*p* < 0.001, χ^2^ test). **In panel B**, the domain-specific completeness of reporting (calculated using the majority rule) is visually considered as high (green), moderate (yellow) or low (red) quality if it scores >60%, between 40% and 60%, or <40%, respectively^15^ (more methodological details in the text). All data are expressed as dimensionless variables.

## Discussion

We assessed 11 European guidelines on the use of TH for neonates, all of which had drawbacks in terms of quality and completeness of reporting, confirming our hypothesis. In terms of the AGREE-II assessment, the German guidelines (which scored the highest and were considered of medium quality) were the best, although no guideline received a good score in the third AGREE-II domain (i.e. rigour of development). Regarding the RIGHT appraisal, none of the guidelines demonstrated satisfactory global completeness of reporting. However, the French guidelines achieved the highest domain-based completeness score.

These are novel and interesting results, since they highlight, for the first time, the downsides and areas of improvement of guidelines about neonatal TH. This is clinically relevant because TH is one of the few evidence-based innovations in neonatology,^2^ and allows to significantly improve mortality and morbidity.^1^ Furthermore it would be important to have high-quality clinical practice guidelines in order to guide practitioners as the patients treated in ‘real life’ may present slightly different situations compared to those enrolled in research projects and because some guidance is anyway needed when adequate evidence lacks. Nevertheless, the reviewed guidelines were not produced with the quality required by the methodology recently recommended in adult critical care medicine.^20^ Of particular concern is the fact that no guidelines achieved a high score in the ‘rigour of development’ and ‘appraised evidence’ domains. These domains are considered the most important parts of the AGREE-II and RIGHT appraisals, respectively.^16^^,^^21^ Therefore, regardless of the overall evaluation and thresholds used,^21^ none of these guidelines should be recommended or applied directly without critical appraisal and modification. These findings highlight that TH risks to be trivialised by guidelines that introduce misleading or unnecessary details that anyway were never tested in a dedicated randomised trial.

TH is an invasive, expensive and complex intervention that shall be carefully applied to all neonates in need but avoided in those who do not fully qualify for the treatment. In fact, implementing low-quality guidelines may result in patients being treated in ways that contradict the available evidence, potentially leading to both overtreatment and undertreatment. This can have negative public health consequences, such as increased costs and an inability to access NICU beds. It can also give rise to medico-legal issues and differences between centres and countries that are not justified by the available evidence.

The American Academy of Paediatrics recently issued an official report stating that neonates offered TH should meet inclusion criteria outlined in the published clinical trials.^22^ Nonetheless, the reviewed guidelines show discrepancies particularly in the eligibility criteria for TH (Supplementary Table S2), which are not entirely supported by the evidence since the original trials that demonstrated the effectiveness of TH used essentially similar criteria.^1^ It seems that, subsequently, local interpretation and different weight of the criteria have brought to different recommendations that still warrant supporting evidence.^3^

Conducting randomised trials in neonatal critical care is known to be difficult, and this has probably also been reflected in the differences about the use of TH in special cases, such as in late preterm patients or those admitted after the first 6 h of life (Supplementary Table S3). The lack of satisfactory score for third AGREE-II domain (i.e. ‘rigour of development’) is also concerning when it comes to these special cases. For example, recent data suggest that cooling late preterm patients may be associated with white matter injury^23^, ^24^, ^25^ and increased likelihood of death/disability.^26^ Furthermore, depending on the local geography and organisation of care, outborn neonates may often reach a referral centre after 6 h of life,^27^^,^^28^ but TH may still be beneficial in these patients.^29^ More rigour is also needed in terms of monitoring and general patient management which were also heterogeneous amongst the reviewed guidelines (Supplementary Table S4). This heterogeneity reflects the lack of dedicated studies about the monitoring, sedation and vital support of these patients. Nonetheless, while the lack of studies is not a fault of guidelines, these latter should anyway provide some pathophysiology-based guidance on the general vital monitoring and support. These latter can significantly impact outcomes for all patients by influencing cardio-respiratory or metabolic function. Furthermore, patients requiring TH may range from those with moderate HIE and no other organ involvement, to those with severe HIE and/or multiple organ failure. Clearly, vital monitoring and intensive care management should be based on pathophysiology and tailored to clinical severity, with closer monitoring and more interventions dedicated to the sickest patients, as it has been recommended for paediatric and neonatal respiratory failure, for example.^30^ Nonetheless, the reviewed TH guidelines do not consider this issue.

The reviewed guidelines, which were all relatively recent (i.e. issued from 2010 to 2020), were essentially based on the same clinical evidence,^1^^,^^3^ and all the guidelines are currently in use, with only one (the French guideline) being under revision. Therefore, we were unable to detect any temporal changes in quality or completeness. Our results should be useful for clinicians, researchers and policymakers who are trying to harmonise practices in Europe by creating guidelines more adherent to available evidence and known pathophysiology. This may be done by national, or even better, continental, organisations by applying strict criteria already used in adult critical care medicine.^20^ This would reduce differences amongst jurisdictions and also help to focus on grey area where higher-quality evidence is needed. Thus, our work may be helpful to design studies that investigate clinically relevant areas which are not currently addressed by the guidelines due to insufficient evidence (e.g. application of TH in special cases or patient monitoring during TH).

Our findings may especially be useful for the end-users, that is the NICU clinicians, enabling them to critically appraise the guidelines and make more informed choices in some borderline cases, while advocating for more rigorous, science-based neonatal intensive care, as recently recommended.^2^ There is an urgent need for these improvements since there are relevant variations across European NICUs in the ‘real-world’ TH application and management.^31^^,^^32^

Our work has several strengths and limitations. We applied a rigorous methodology based on the two most accepted tools, AGREE-II and RIGHT, by a large multidisciplinary panel, representing various professionals who deal with TH in clinical practice. The evaluation process was blind and anonymous so no panellist could have influenced any other. We analysed a convenient sample of European guidelines from 11 countries representing the majority of ESPNIC membership. However, we acknowledge that the sample did not include the guidelines of all European countries. As ESPNIC membership is unevenly distributed across Europe, some countries cannot be represented as they have no ESPNIC members. This is an inherent limitation of the work. In Europe, healthcare is regulated at the country level, thus several issues are not harmonised: this may have contributed to some differences amongst the guidelines through differently organised settings, reimbursement policies and regionalisation of care. The reviewed guidelines were, in most cases, not published in peer-reviewed, academic journals and this could have partially contributed to their generally moderate-low quality. None of them addressed patients under extracorporeal life support, thus we could not evaluate this special circumstance. Our results cannot be translated to guidelines from non-European countries, which we have not evaluated, and that can reflect different healthcare systems, birth rate, resources allocation and regionalisation of care.

In conclusion, in a sample of 11 European countries, guidelines for neonatal TH have an insufficient quality and completeness of reporting with several aspects to be improved. The German guidelines scored best for quality but a good score in the rigour of development was not achieved by any guideline. No guideline showed satisfactory global completeness of reporting. However, the French guidelines scored the highest domain-based completeness. Our findings show that applying these guidelines without a critical appraisal might lead to under- and over-treatment or induce potentially unsafe decisions in special cases (e.g. late preterm patients or neonates beyond the first 6 h of life) and about the general critical care of these patients. Given these results no guideline can be recommended to be directly applied without critical appraisal and modifications: future guidelines should consider these findings and the recommendations issued in adult critical care to improve their quality.

## Contributors

Prof. De Luca conceived the manuscript, wrote the first draft and collected the literature information, analysed the data and participated to the voting. Dr. Neri collected the literature information, analysed the data and participated to the voting. Prof. De Luca and Dr. Neri verified the data. Drs. Alonso-Ojembarrena, Ippolito, Milan, Zanin Schettler and Profs. Piastra and Ghi participated to the voting and interpreted the data. Prof. Sanchez-Luna helped in the general study management, literature search and data interpretation. Prof. Cortegiani supervised the whole study, interpreted the data and helped in their analysis and verified the data. All authors critically reviewed the manuscript, contributed important intellectual content, approved the final version of the manuscript and accept responsibility for its submission.

## Data sharing statement

The anonymised datasets will be available upon adequate request for research purposes, respecting all relevant regulations and with the establishment of confidential data transfer agreements.

## Declaration of interests

Prof. De Luca received speaker or scientific consulting fee or research assistance from Airway Therapeutics, Oak Hill Bio, Chiesi Farmaceutici, Medtronic, Getinge, Zoll, Radiometer, Mediprema all completely unrelated to the field of hypothermia and this work. All other authors have no conflict of interest to declare.
