# Supplementary material for: Clinical practice guidelines for neonatal therapeutic hypothermia across European Society for Paediatric and Neonatal Intensive Care (ESPNIC) member countries: a multidisciplinary blind comparison
Source: Lancet Reg Health Eur. 2026 Sep 22;71:101874. doi: 10.1016/j.lanepe.2026.101874 (PMC13652852; doi:10.1016/j.lanepe.2026.101874)
Supplement: Supplementary file 1 [file mmc1.docx]

**ONLINE SUPPLEMENT**

**for**

**Evidence – based multidisciplinary blind comparison of European clinical practice guidelines for neonatal therapeutic hypothermia**

*Prof.* Daniele De Luca (PhD),^1,2^ Costanza Neri (MD),^3^

Almudena Alonso-Ojembarrena (MD, PhD),^4,5^ Mariachiara Ippolito (MD),^6,7^

Anna Milan (PhD),^8^ *Prof.* Marco Piastra (MD),^9^ *Prof.* Manuel Sanchez-Luna (PhD),^10^

Karl F. Schettler (MD),^11^ Anna Zanin (PhD),^12^ *Prof.* Tullio Ghi (PhD),^13^

and *Prof.* Andrea Cortegiani (MD)^6,7^

on behalf of the Neonatal Critical Care Section of the European Society for Pediatric and Neonatal Intensive Care

*1 Division of Pediatrics and Neonatal Critical Care, “A. Béclère” Medical Centre,*

*Paris Saclay University Hospital, APHP - Paris, France*

*2 Physiopathology and Therapeutic Innovation Unit, INSERM U999 - Paris Saclay University - Paris, France*

*3 School of Pediatrics, University of Chieti-Pescara “G.D’Annunzio”*

*4 Neonatal Intensive Care Unit, Puerta del Mar University Hospital - Cádiz, Spain*

*5 Biomedical Research and Innovation Institute of Cádiz (INiBICA), Research Unit, Puerta del Mar University Hospital - Cádiz, Spain*

*6* *Dept. of Precision Medicine in Medical, Surgical and Critical Care (Me.Pre.C.C), University of Palermo - Palermo, Italy*

*7 Dept. of Anaesthesia, Intensive Care and Emergency, Policlinico Paolo Giaccone - Palermo, Italy*

*8 Neonatal Unit, Evelina London Chidren’s Hospital-Guy’s & St.Thomas’ Hospital - London, UK*

*9 Dept. of Biotechnology, Critical and Perioperative Medicine, Catholic University of the Sacred Heart - Rome, Italy*

*10 Division of Neonatology, “Gregorio Marañon” University Hospital, Universidad Complutense - Madrid, Spain*

*11 Children Hospital St. Marien, LA-Regio-Kliniken Landshut,*

*Teaching Affiliate of the Ludwig-Maximilians-University of Munich – Munich, Germany*

*12 Dept. of Women, Children and Adolescent, Hôpitaux Universitaires Genève and University of Geneve - Geneve, Switzerland*

*13 Dept. of Woman and Infant Sciences Health, Catholic University of Sacred Heart - Rome, Italy*

**Supplementary Table 1. Details of the panel composition**… Pg.2

**Supplementary Table 2. Criteria to start therapeutic hypothermia**… Pg.3-6

**Supplementary Table 3. Special cases where therapeutic hypothermia has been proposed “off label”**… Pg.7-9

**Supplementary Table 4. General NICU management during therapeutic hypothermia…** Pg.10-12

**Supplementary Table 5. AGREE-II qualitative comments**… Pg.13-16

**Supplementary Table 1. Details of the panel composition.** Panelists are indicated by their initials in alphabetic order. *The methodologist supervised the AGREE-II and RIGHT-II evaluations but did not vote for the guidelines evaluation. ^#^Indicates panelists holding a position as (tenured) professors. The Chair of ESPNIC Neonatal Critical Care Section (MSL) is not listed here because he was not part of the panel and did not vote for the guidelines evaluation, but was eligible for the authorship. All raters, but the obstetricians, had direct experience in the clinical use of therapeutic hypothermia. **Abbreviations**: ESPNIC: European Society for Pediatric and Neonatal Intensive Care.

|  | **Country** | **Specialty** | **Years of**  **clinical experience** | **Gender** | **ESPNIC member** | **Notes** |
| --- | --- | --- | --- | --- | --- | --- |
| **Co-chairs** | | | | | |  |
| AC | Italy | Methodology | >10^#^ | M | No | Co-chair* |
| DDL | France | Neonatology | >10^#^ | M | Yes | Co-chair |
| **Core group** | | | | | | |
| AAO | Spain | Neonatology | >10 | F | Yes |  |
| AM | UK | Neonatology | >10 | F | Yes |  |
| AZ | Switzerland | Neonatology | >10 | F | Yes |  |
| CN | Italy | Neonatology | <2 | F | Yes | Trainee representative |
| KS | Germany | Neonatology | >10 | M | Yes |  |
| **Additional multi-disciplinary panelists** | | | | | |  |
| MCI | Italy | Adult critical care | >10 | F | No |  |
| MP | Italy | Ped critical care | >10^#^ | M | Yes |  |
| TG | Italy | Obstetrics | >10^#^ | M | No |  |

**Supplementary Table 2. Criteria to start therapeutic hypothermia.** Data were independently extracted by two reviewers and cross-verified (more details in the main text). **Abbreviations**: aEEG: amplitude-integrated EEG; BW: birth weight; GA: gestational age.

| **Country** | **Baseline**  **characteristics** | **Criteria A** | **Criteria B** | **Criteria C** |
| --- | --- | --- | --- | --- |
| France | - ≥ 36 weeks’ GA AND - > 1800 g AND - ≤ 6 hours postnatal age | **At least one of the following:**   - Umbilical cord or any blood sample within 1 hour of life showing:   - pH ≤7.00 OR   - Base Deficit ≥ 16 mmol/L OR   - Lactate ≥ 11 mmol/l OR - Apgar score of ≤5 at 10’ after birth OR - Continued need for resuscitation, including endotracheal or mask ventilation, at 10’ after birth | - Moderate to severe encephalopathy defined by Sarnat & Sarnat (Stage II or III):^1^   - Consciousness alteration (lethargy, stupor, coma)   AND   - **At least one** of the six listed items:   1) Gross motor activity: reduced or stereotyped movements/ absent or decorticate-like tremors.   2) Posture: limb extension or distal flexion/ decorticate or abnormal upper limb flexion   3) Tone: hypotonia or clenched hands and feet/flaccidity   4) Suction: weak or chewing/ absent   5) Autonomic system (pupils, heart rate, respiration, brainstem reflexes): constricted/bradycardia/periodic breathing/present reflexes or fixed dilated/unchanging HR/apnea/absent reflexes)   6) Clinical seizures - If aEEG/EEG is not available before the onset of TH, treatment must be started according only to the clinical signs:   - Consciousness alteration (lethargy, stupor, coma)   AND   - **At least two** of the six listed items | If aEEG/EEG can be done before TH, clinical neurological signs AND aEEG/EEG abnormalities must be present:   - aEEG abnormalities: - upper margin >10 µV, lower margin <5 µV - both margins <10 µV and <5 µV - seizures - EEG abnormalities: - worsening trace (inactive, paroxysmal, or theta- poor) - intermediate trace (discontinuous) - seizures |
| Germany | - ≥ 36 weeks’ GA AND - ≤ 6 hours postnatal age | **At least one of the following:**   - Umbilical cord or any blood sample showing:   - pH ≤ 7.00 OR  - Base Deficit ≥ 16 mmol/L If blood gas analysis is unavailable, additional criteria are required:   - clinical evidence of fetal hypoxia (e.g., late or variable decelerations, cord prolapse or avulsion, uterine rupture, maternal trauma or haemorrhage, or maternal cardiac arrest) AND/OR - Apgar score ≤ 5 at 10’ OR need for ventilation/resuscitation for at - least 10‘ after birth | - Moderate to severe encephalopathy defined by **at least three pathological signs within the six categories** according to modified Sarnat score (Stage II or III)^1^ | - aEEG showing: - moderately abnormal activity (upper margin >10 µV, lower margin <5 µV) OR - severely abnormal activity (both margins <10 µV and <5 µV) OR - burst suppression |
| Italy | - > 35 weeks’*** GA AND - BW > 1800 g AND - < 6 hours postnatal age   **** According to a recent systematic review^7^, aEEG screening may be considered for 35-week infants meeting asphyxia criteria, whereas TH should be reserved for carefully selected cases.* | - Apgar Score ≤ 5 at 10’ OR - Intubation OR continued need for respiratory support at 10’ OR - Fetal or neonatal acidosis defined as:   - pH≤ 7.0 OR   - Base deficit ≥ 12 mEq/l* observed on umbilical artery blood gas analysis or on any blood gas within the first 60’   **If umbilical cord flow is interrupted (e.g., shoulder dystocia), cord blood gas is considered falsely normal, so a neonatal blood gas is required.* | - **At least two clinical signs between 30 and 60‘ of life** according to modified Sarnat score^1^: **   - Consciousness alteration (lethargy***/stupor, coma)   - Spontaneous movements: reduced/absent   - Posture: distal flection or complete extension/decorticate   - Tone: hypotonia/flaccidity   - Primitive reflexes (Moro and suction): weak or incomplete/absent   - Pupillary abnormalities: miosis/mydriasis/absent reactivity or gaze deviation   - Clinical seizures   ***In case two of these two signs are present but there is no criterion A (i.e. no signs of perinatal asphyxia), the guideline demands to perform an EEG/aEEG anyway*  **** According to a recent systematic review^7^,* *the Sarnat definition of “lethargy” has limited reliability and low sensitivity (43%). Therefore, Criterion B is defined as the presence of at least two neurological signs to maximize sensitivity,* *rather than using altered consciousness as a mandatory criterion.* | - **At least one** of the following abnormal aEEG patterns after 30’ of recording*** before administering antiepileptic, analgesic, or sedative therapy:   - Moderately abnormal activity (upper margin >10 µV, lower margin <5 µV)   - Severely abnormal activity (both margins <10 µV and <5 µV)   - Electrical seizures   OR   - **At least one** of the following abnormal patterns on conventional EEG:   - Moderate abnormalities: persistently discontinuous background with IBI   <10 s, unclear sleep-wake cycles   - Severe abnormalities: discontinuous background with IBI 10–60 s, amplitude <10 µV, absent sleep- wake cycles - Very severe abnormalities: inactive trace or IBI >60 s - Electrical seizures   ****According to a recent systematic review^7^, Future guidelines may recommend six-hour aEEG monitoring to detect delayed changes.* |
| Netherlands | - ≥ 35 weeks’ GA AND - > 1800 g AND - < 6 hours postnatal age | **At least one** of the following:   - Apgar score of ≤ 5 at 5‘after birth - Need for resuscitation - Ongoing need for PPV at 10’ after   birth   - Umbilical cord or any blood   sample within 1 hour of life showing:   - pH <7.00 OR - Base Deficit < -16 mmol/L OR - Lactate >10 mmol/l | Thompson Score^4^ ≥ 7 at 1 hour after resuscitation | - Abnormal aEEG:   - Discontinuous normal voltage pattern (DNV): upper margin >10 µV, lower margin <5 µV   - Burst suppression (BS)   - Continuous low voltage (CLV): continuous background ≤5 µV   - Inactive flat trace (FT): isoelectric tracing with background < 5 µV   - Seizures   **Therapeutic hypothermia is initiated when either the Thompson score ^4^ OR the aEEG meets the specified criteria* |
| Norway | - ≥ 36 weeks’ GA AND - < 6 hours postnatal age | **At least one** of the following:   - Apgar score of ≤ 5 at 10‘after birth - Ongoing need for PPV at 10’ after   birth   - Umbilical cord or any blood   sample within 1 hour of life showing:   - pH <7.00 OR - Base Deficit ≤ -16 mmol/L | Decreased level of consciousness (drowsiness/lethargy, obtundation/stupor, or unconsciousness/coma) with **at least one** of the following symptoms according to modified Sarnat score^1^:   - Muscle hypotonia - Abnormal deep tendon reflexes - Weak or absent sucking reflex - Seizure | - Abnormal aEEG:   - Normal background activity with occasional   - epileptic activity.   - Moderately abnormal background activity   - (upper margin >10 µV and lower margin <5 µV)   - Severely abnormal background activity   - (upper margin <10 µV and lower margin <5 µV)   - Burst suppression pattern.   - Continuous seizure activity |
| Portugal | - ≥ 36 weeks’ GA AND - < 6 hours postnatal age | - Apgar Score ≤ 5 at 10’ - Need for resuscitation10’ - Umbilical cord or any blood   sample within the first hour of life showing neonatal acidosis defined as:   - pH <7.00 OR - Base Deficit < -16 mmol/L | - Thompson Score^4^ ≥ 7 at 1 hour after resuscitation | - The presence of aEEG abnormalities reinforces the indication for TH:   - seizures   - moderately abnormal or discontinuous background trace (upper margin >10 µV, lower margin <5 µV)   - burst-suppression pattern   - suppressed trace (flat trace)   - electrical status epilepticus defined as a continuous or rapidly recurring seizure with a sustained electrical band > 10 µV   **If clinical criteria suggest a severe insult (severe acidosis or a very low Apgar score), a normal aEEG should not rule out hypothermia treatment.* |
| Slovakia | - ≥ 35 weeks’ GA AND - ≥ 1800 g AND - < 6 hours postnatal age | **At least one** of the following:   - pH < 7.0 OR BE ≥ 16 mmol/L AND/OR lactate > 10 mmol/L in umbilical cord blood gas analysis or blood sample obtained from the infant within 1 hour of birth - Apgar score < 6 at 10‘ OR ongoing need for resuscitation PPV after 10‘ - Sentinel intrapartum events (e.g., umbilical cord prolapse, placental abruption, uterine rupture, severe fetal heart rate abnormalities, prolonged shoulder dystocia, severe fetal blood loss such as vasa previa) | - Clinical seizures OR - Thompson score^4^ ≥ 7 points OR - Two or more moderate or severe signs of HIE according to the modified Sarnat classification^1^ with abnormal aEEG confirmation OR - At least three moderate or severe signs of HIE according to the modified Sarnat classification^1^ without requiring aEEG confirmation OR - SIBEN neurological score^6^ indicating moderate/severe HIE   ** If the infant meets criteria A and B, TH can be initiated.* | - Abnormal aEEG findings after 30’ of recording:   - Seizures   - Moderately abnormal amplitude (upper margin > 10 μV, lower margin ≤ 5 μV)   - Suppressed amplitude (upper margin < 10 μV, lower margin < 5 μV)   **aEEG is required in borderline cases (2 Sarnat moderate/severe signs). When ≥3 moderate/severe Sarnat signs are present, clinical criteria alone are sufficient.* |
| Spain | - ≥ 35 weeks’ GA AND - > 1800 g AND - < 6 hours postnatal age | - Apgar score of ≤5 at 5’ after birth AND/OR - Umbilical cord blood sample showing:   - pH ≤ 7.00   - Base deficit > 16 mmol/L | - Moderate to severe encephalopathy defined by García-Alix score^2,3^ | - aEEG recording showing   - moderately/severely abnormal activity   - presence/absence of sleep-wake cycle presence/absence of seizures |
| Switzerland | - > 35 weeks’ AND - < 6 hours postnatal age AND - BW ≥ 2000 g | **At least two of the following**:   - Apgar score ≤ 5 at 10’ after birth - Continued need for resuscitation, including endotracheal or mask ventilation, at 10‘after birth - Any occurrence of umbilical cord, arterial or capillary within 60’ of birth showing:   - pH ≤ 7.00 OR   - Base deficit ≥ 16 mmol/L OR   - Lactate ≥ 12 mmol/L | - Clinical seizures OR - Moderate to severe encephalopathy defined by Sarnat (Stage II or III)^1^ OR Thompson Score^4^ ≥7 | - aEEG findings according to the classification of de Vries et al^5^ can aid hypothermia decisions but should not delay treatment:   - Continuous normal voltage pattern: continuous activity with minimum amplitude ~5–10 µV and maximum ~10– 25 µV   - Discontinuous normal voltage pattern: discontinuous background with minimum <5 µV and maximum >10 µV   - Burst suppression with minimum 0–1(2) µV, bursts >25 µV   - Continuous low voltage: continuous background ≤5 µV)   - Inactive flat trace: isoelectric tracing with background < 5 µV |
| Turkey | - ≥ 36 weeks’ GA AND - < 6 hours postnatal age AND - > 1800 g | - Apgar score < 5 at 10’ after birth OR continued need for resuscitation - Any occurrence of umbilical cord, arterial or capillary within 60’ of birth showing:   - pH ≤7.00 OR   - Base Excess ≤ - 16 mmol/l   **Initiate TH in infants with non-qualifying pH or BE values if both additional criteria are present (low Apgar score and clinical encephalopathy).* | - Moderate to severe encephalopathy defined by Sarnat (Stage II or III)^1^ OR Thompson Score^4^ ≥7 | - Abnormal aEEG findings after 30’ of recording:   - Continuous normal voltage (lower margin 7–10 µV, upper margin 10–25 µV)   - Discontinuous normal voltage (lower margin < 5 µV, upper margin > 10 µV)   - Burst suppression (stable lower amplitude of 0–1 µV and bursts with amplitudes >25 µV)   - Consistently low voltage (around or below 5 µV)   - No activity, flat line (inactive background below 5 µV) |
| UK | - ≥ 36 weeks’ GA AND - < 6 hours postnatal age | **At least one of the following**:   - Apgar score ≤ 5 at 10’ after birth OR - Continued need for resuscitation, including endotracheal or mask ventilation, at 10’ after birth OR - Acidosis defined as   - pH ≤ 7.00  - Base deficit ≥ 16 mmol/l   - in any cord or baby gas sample within 60’ of birth | Moderate to severe encephalopathy defined by modified Sarnat score (Stage II or III)^1^:   - Altered state of consciousness (lethargy, irritable/stupor or coma) AND - **At least one of the following** according to the modified Sarnat scoring system: - Spontaneous activity (decreased or no activity) - Posture (distal flexion, full extension, frog-legged position or decerebrate) - Tone (hypotonia or flaccid) - Primitive reflexes (suck, Moro): weak/incomplete or absent - Autonomic system (pupils, heart rate, respiration): constricted/bradycardia/periodic breathing or skew gaze non-reactive to light, fixed dilated/variable HR, irregular HR/apnea) - Clinical seizures | - At least 30‘ of aEEG recording showing one of the following:   - normal background with some seizure activity   - moderately abnormal activity   - suppressed activity   - continuous seizure activity   **Inability to obtain aEEG should not prevent or delay treatment if there is evidence from A and B criteria.* |

**References**

1. Sarnat HB, Sarnat MS. Neonatal encephalopathy following fetal distress. A clinical and electroencephalographic study. *Arch Neurol* 1976;**33**(10):696–705.

2. Garcia-Alix A, Arnaez J, Arca G et al. Development, reliability and testing of a new rating scale for neonatal encephalopathy. *J Pediatr* 2021; **235**: 83-91.e7

3. Garcia-Alix A, Arnaez J, Neurologia neonatal de un vistazo. Ed. Cabeza de Chorlito (Madrid, Spain) 2022, pg.110

4. Thompson CM, Puterman AS, Linley LL, et al. The value of a scoring system for hypoxic-ischaemic encephalopathy in predicting neurodevelopmental outcome. *Acta Paediatr* 1997;**86**(7):757–761

5. de Vries LS, Hellstrom-Westas L. Role of cerebral function monitoring in the newborn. *Arch Dis Child Fetal Neonatal Ed* 2005; **90**(3): F201-207

6. Perez JM, Golombek SG, Sola A. Clinical hypoxic-ischemic encephalopathy score of the Iberoamerican Society of Neonatology (Siben): A new proposal for diagnosis and management. *Rev Assoc Med Bras* (1992). 2017 Jan 1;**63**(1):64-69.

7. Ancora G, Gallini F, Fumagalli M, et al. Therapeutic hypothermia in newborns: evidence-based guidelines from a systematic review. *Ital J Pediatr*. 2026 Apr 27;**52**(1):103.

**Supplementary Table 3. Special cases where therapeutic hypothermia has been proposed “off label”.** Data were independently extracted by two reviewers and cross-verified (more details in the main text). **Abbreviations**: aEEG: amplitude-integrated EEG; BW: birth weight; ECMO: extracorporeal membrane oxygenation; GA: gestational age; HIE: hypoxic-ischemic encephalopathy; N/A: not available; SUPC: sudden unexpected postnatal collapse; TH; therapeutic hypothermia.

| **Country** | **Late hypothermia**  **(>6h postnatal age)** | **Special circumstances** | **ECMO patients** |
| --- | --- | --- | --- |
| France | N/A | N/A | N/A |
| Germany | N/A | N/A | N/A |
| Italy | - > 35 weeks’ GA AND - BW > 1800 g AND - 6 - 24 hours postnatal age meeting criteria A and B even without evidence of aEEG/EEG abnormalities (criteria C) are eligible for TH for 96 hours***   **** According to a recent systematic review^7^, a postnatal age >6 hours weakens the recommendation, with very low certainty of evidence in infants born <36 weeks’ GA.* | - Cooling is recommended for neonates > 35 weeks’ GA AND > 1800 g AND < 6 hours postnatal age meeting criteria B and C without evidence of perinatal asphyxia (absent criteria A) - Cooling is recommended for outborn neonates referred to a tertiary care center fort TH meeting criteria A and C but with normal neurological evaluation within the first 6 hours of life - SUPC AND > 35 weeks’ GA AND BW > 1800 g AND < 6 hours from the event meeting criteria A (only blood gas data or the need for respiratory support), B and C | N/A |
| Netherlands | In exceptional cases (i.e., logistical delays or late-onset clinical signs), cooling may start a few hours beyond the 6-hour window | Contraindicated if:   - BW < 1800 g OR - Lethal congenital anomalies   Special cases defined as follows:   - If Thompson score is 5 at 1 hour of life and rises afterward, consider referral for observation and aEEG. - If the Thompson score is ≥ 7 initially but drops < 7 within the first hour of life, cooling is not needed if aEEG is normal. - If the score remains ≥ 7 after the first hour of life, therapeutic hypothermia should be started and continued, even if the later aEEG appears normal. | N/A |
| Norway | Infants aged 6–12 hours after birth and those requiring early surgery should be considered for TH only after an individualized risk–benefit assessment | Contraindicated if:   - Major intracranial hemorrhage, other severe bleeding, severe uncorrectable coagulopathy - Central nervous system malformations or chromosomal abnormalities with limited life expectancy - Respiratory or hemodynamic instability - Major congenital anomalies requiring surgery within the first days of life - GA 33-35 weeks’   Special cases defined as follows   - Criterion B uncertain with abnormal aEEG: TH is recommended. - Criterion B uncertain with normal aEEG: withholding TH may be considered. | N/A |
| Portugal | Delayed admission (>12 hours of age) or initiation of passive cooling after 6 hours represent relative contraindications. TH may be considered after discussion with the treating unit and the parents. | Special cases defined below represent only a relative contraindication. The initiation of treatment be considered only after discussion with the unit and the parents:   - Intrauterine growth restriction - GA < 36 weeks’ - Major congenital malformations - Need for surgery within the first 3 days of life - Major intracranial hemorrhage - Postnatal cardiorespiratory arrest - Highly unstable newborns   Upon admission to the treatment center, for an outborn infant: reassessment using the Thompson score; passive hypothermia; aEEG (at least 6 hours).  In cases of mild or moderate HIE (i.e., Thompson score <7), the infant may be monitored up to 12 hours of age maintaining passive hypothermia to allow for a therapeutic decision based on more data.  Withholding treatment should only be considered if the infant has borderline asphyxia criteria, a Thompson score ≤ 7, and a normal aEEG. | N/A |
| Slovakia | Starting TH after 6 hours may be considered on an individual basis, with possible initiation up to 12–24 hours after birth | **Absolute contraindications**:   - GA <35 weeks - BW <1800 g - Mild HIE - Planned palliative care (moribund infants or those with severe congenital anomalies/genetic abnormalities) - Severe coagulopathy and/or active bleeding - Severe head trauma or intracranial hemorrhage   **Relative contraindications**:   - In case of suspected coagulopathy, partial passive cooling may be considered to maintain an axillary/rectal temperature of 34.5°C while awaiting coagulation results - PPHN alone is not a contraindication - Refractory hypoxemia despite maximal therapy (e.g., FiO2 >80%) - In case of PPHN and meconium aspiration syndrome, decisions should be guided by clinical status and echocardiography | N/A |
| Spain | Consider compassionate use if 6-12 hours postnatal age | Consider compassionate use if:   - 34-35 weeks’ GA OR - BW < 1800 g | N/A |
| Switzerland | Contraindicated in infants > 6 hours old | Contraindicated if < 35 weeks GA | N/A |
| Turkey | TH beyond 6 hours after birth should be considered only after an individualized risk–benefit assessment and family counselling. | Contraindicated if:   - GA <34 weeks’ - BW < 2000 g - Suspicion of an alternative cause (e.g., inherited metabolic disorders or relevant family history) - Severe comorbidities (e.g., severe head trauma, extensive intracranial haemorrhage or life-threatening coagulopathy) - Maternal chorioamnionitis, trisomy 13 or trisomy 18, multiorgan congenital abnormalities - Infants meeting TH criteria but with maternal chorioamnionitis or severe multiorgan dysfunction may not be appropriate candidates and require individualized risk–benefit assessment. - Special cases that do not meet the standard diagnostic criteria for TH (i.e., 34–35 weeks' GA infants, with umbilical cord blood pH >7, and borderline acid-base values) should be considered only after an individualized risk–benefit assessment and family counselling. - Routine cooling of infants with stage I HIE is not recommended. | N/A |
| UK | In the absence of harm or alternative treatments, infants diagnosed with presumed HIE between 6 and 24 hours of age may still benefit from TH | - Without evidence of benefit, TH for mild HIE is not recommended outside clinical trials. - Following animal data on TH effectiveness after postnatal HIE, TH may be considered in infants with moderate/severe HIE after SUPC, with a second senior opinion and parental consent. | N/A |

**Supplementary Table 4. General NICU management during therapeutic hypothermia.** Data were independently extracted by two reviewers and cross-verified (more details in the main text). **Abbreviations**: aEEG: amplitude-integrated EEG; N/A: not available; NIRS: near-infrared spectroscopy; TH; therapeutic hypothermia.

| **Country** | **Mechanical**  **Ventilation** | **Sedation** | **Central Venous**  **Access** | **Arterial Access** | **NIRS** | **aEEG** | **Fluid**  **restriction** | **Sodium control** |
| --- | --- | --- | --- | --- | --- | --- | --- | --- |
| France | Recommended | Analgesia and sedation with opioids are recommended | Recommended | N/A | N/A | Recommended during cooling and rewarming | Recommended | Not indicated |
| Germany | If respiratory instability (i.e., apneas), provide respiratory support  to prevent cerebral hypoxia | Analgesia and sedation with opioids are recommended | N/A | N/A | N/A | Recommended during cooling and rewarming | N/A | N/A |
| Italy | Indicated in cases of respiratory failure, persistent pulmonary hypertension, or seizures with apnea | - Fentanyl loading dose of 1–2 µg/kg over 1 hour, followed by continuous infusion of 0.5–1 µg/kg/h - Continuous morphine ≤ 10 µg/kg/h - Dexmedetomidine 0.2–0.5 µg/kg/h either as monotherapy or in addition to opioids*****   ****Highly recommended according to a recent systematic review^7^* | Recommended | Useful but not strictly recommended | N/A | Recommended during hypothermia, rewarming, and 4–  12 hours after | Initial maintenance fluids at 60 ml/kg/day | Restrict free water intake rather than give sodium chloride to avoid severe hyponatremia |
| Netherlands | N/A | Analgesia and sedation with morphine are recommended (also in spontaneously breathing infants) | Recommended (double lumen) | Recommended | Recommended | Recommended during cooling until 24 hours after rewarming | N/A | N/A |
| Norway | Most cooled infants require intubation, as adequate analgesia may be difficult to achieve in spontaneously breathing infants. Conventional mechanical ventilation, HFOV, and/or iNO may be used as clinically indicated. | - Morphine loading dose 100 µg/kg/hour for 2 hours followed by maintenance infusion of 20 µg/kg/hour (range 5–40 µg/kg/hour) - Fentanyl loading dose 1–3 µg/kg as an IV bolus followed by maintenance infusion of 2 µg/kg/hour (range: 0.5–2 µg/kg/hour) - Clonidine 0.5–2.0 µg/kg/hour. - Dexmedetomidine 0.2–1.0 µg/kg/hour - Midazolam 10–50 µg/kg/hour | Recommended (double lumen) | Recommended | N/A | Recommended during cooling and rewarming | Fluid intake 40–60 mL/kg/day.  In case of renal failure, fluid restriction is recommended (30 mL/kg/day plus measured fluid losses). | N/A |
| Portugal | Invasive ventilation is not mandatory but is usually required in moderate to severe HIE to ensure adequate oxygenation. When used, volume-guaranteed modes are preferred | - Morphine (initiated at 50 µg/kg, followed by a continuous infusion of 5–10 µg/kg/h) - Low-dose dexmedetomidine (0.05–0.5 µg/kg/h) - Midazolam (10–60 µg/kg/h) without neglecting risks of hemodynamic adverse effects and impaired neuronal/synaptic development. - Chloral hydrate (50 mg/kg PO) can occasionally be considered as a benzodiazepine alternative. Contraindicated in case of cardiac, renal, or hepatic dysfunction | Recommended (double lumen) | Recommended | Recommended | Recommended during cooling until 24 hours after rewarming | Initial maintenance fluids at 40–60 mL/kg/day.  Later adjust according to body weight, fluid balance, urine output, electrolytes (sodium/chloride). Aim for a urine specific gravity of 1.010 and a serum osmolality of 290 mOsm/L. | Maintain sodium levels at the upper limit of the normal range |
| Slovakia | Use spontaneous breathing, NIV or MV (CMV/HFO/NO) as clinically required | - Opioids (e.g., morphine, fentanyl) - Alpha-2 adrenergic receptor agonists (e.g., dexmedetomidine, clonidine) as an alternative to opioids - Benzodiazepines (e.g., lorazepam, midazolam) should not be routinely used | Recommended | N/A | Recommended | Recommended during cooling and rewarming for up to 96 hours | Fluid intake: 40–60 ml/kg/day during the first day.Later adjust according to circulation, natremia, and urine output.  In case of renal failure, fluid restriction is recommended (30 mL/kg/day plus measured fluid losses) | Maintain sodium levels at 135–140 mEq/L |
| Spain | Indicated in cases of respiratory failure | - Fentanyl 1–3 µg/kg/h - Morphine loading dose of 240 µg/kg followed by continuous infusion of 20 µg/kg/h - Dexmedetomidine loading dose 0.4 µg/kg/h followed by continuous infusion of 0,2-0,4 µg/kg/h (max 1-1,5 µg/kg/h) - Avoid midazolam | Recommended | Recommended | Recommended | Recommended during cooling and rewarming | Recommended | N/A |
| Switzerland | Most cooled infants require mechanical ventilation | - Continuous morphine infusion (10-20 µg/kg/hour) could be used - In non-ventilated infants: low doses of continuous morphine (5 -10 µg/kg/hour) or chloral hydrate (20-50 mg/kg) | N/A | Recommended | N/A | Recommended during cooling and rewarming | Initial fluid requirement between 40-60 ml/kg/day | N/A |
| Turkey | Mechanical ventilation may be needed | Analgesia is recommended with:   - morphine 100 µg/kg OR - fentanyl 5 µg/kg | If clinically indicated, catheter placement may be required | N/A | N/A | Recommended during cooling and rewarming for up to 96 hours | Initial fluid requirement between 40-50 ml/kg | In the case of cerebral edema, maintain sodium levels at the upper limit of the normal range |
| UK | Intubation and ventilation should be considered to maintain adequate  gas exchange | - Pain and distress should be treated with opiate medication by continuous infusion of 0,2-0,4 µg/kg/h (max 1- 1,5 µg/kg/h) - Avoid midazolam | Recommended | Recommended | N/A | Recommended during cooling and rewarming | Initial maintenance fluids at 40-60 ml/kg/d | Avoid severe hyponatremia |

**Supplementary Table 5. AGREE-II qualitative comments**. This appendix reports the anonymized free-text comments provided by appraisers, along with the title of each AGREE-II domain to which they refer or the free comments voluntarily left by the rater in general about that guidelines. Raters are identified only by anonymous numerical codes. If a rater or domain is not listed, this means that no comments were given for that rater or domain.

## FRANCE

***The guideline development group includes individuals from all relevant professional groups.***

**Rater 1:** no idea of professional role of authors

**Rater 1:** not clear who the funding body is ( what is GEN-IF?)

**Rater 1:** reported as no conflicts

**Rater 4:** Maternity ward, NICU physicians, and transport teams

**Rater 4:** All recommendations at the same level

**Rater 4:** N/A: No fundings are described in this guideline

**Rater 6:** not reported

**Rater 6:** partially reported

## GERMANY

***The overall objective(s) of the guideline is (are) specifically described.***

**Rater 1:** the aim is described as "reccomendation for the general treatment of babies…" so it's clearly stated even though a weak statement

**Rater 1:** mentioned but not acted on

**Rater 1:** unclear

**Rater 3:** No information

**Rater 4:** Only three authors are listed, but not their affiliation

**Rater 4:** Delphi process

**Rater 4:** Only the date: 2018

**Rater 4:** All recommendations are equal

**Rater 6:** not clear description of the methods

**Rater 6:** unclear benefit of treating subclinical seizures or the lack of data for applying hypothermia to preterm infants.

**Rater 6:** neuroprotective benefits of hypothermia, potential side effects of cooling (e.g., subcutaneous fat necrosis), and the risks associated with the underlying condition (e.g., multi-organ dysfunction).

**Rater 6:** Recommendations are logically placed at the conclusion of relevant sections and often use words like "sollte" (should) or "wird empfohlen" (is recommended). However, they are not visually set apart in summary boxes or bolded lists

**Rater 6:** there is monitoring and not auditing

## ITALY

***The guideline development group includes individuals from all relevant professional groups.***

**Rater 1:** not specified

**Rater 3:** Health intent and population declared, outcome not clearly specified

**Rater 3:** No information

**Rater 4:** NA: not described their affiliations

**Rater 4:** NA: not described

## SPAIN

***The guideline development group includes individuals from all relevant professional groups.***

**Rater 4:** Only neonatologists

**Rater 4:** Unclear

## SWITZERLAND

***There is an explicit link between the recommendations and the supporting evidence.***

**Rater 1:** not always

## UK

***The health question(s) covered by the guideline is (are) specifically described.***

**Rater 1:** brief background description, the concept of Asphyxia and the physiopathology of brain injury are not well described and assumed as known.

**Rater 1:** some about training and audit

**Rater 1:** unclear

**Rater 1:** not mentioned

**Rater 3:** Member of the panel, unclear how preferences were addressed (information not reported)

**Rater 4:** Not described

**Rater 4:** All recommendations are equal

**Rater 6:** Comprehensive reference list (38 citations) but no explicit description of database search or selection criteria

**Rater 6:** No methodology described

**TURKEY**

***Free comments***

**Rater 1:** Detailed conceptual/definitional introduction is given, but no single explicit 'objectives' statement as in some other national guidelines

Health question thoroughly specified via separate diagnostic criteria, treatment criteria and explicit exclusion criteria sections

Population very precisely defined: GA ≥36 wks, ≤6h, pH/BE thresholds, Apgar criteria, encephalopathy severity, plus a detailed list of exclusion criteria (a-f)

Authors/editors are all TND neonatology professors; no evidence of multidisciplinary input (obstetrics, neurology, nursing, family/patient representatives)

No evidence that patient/family views were sought during development (the informed consent form is a clinical-care tool, not a development input)

Target users implied (level-3 NICU neonatology teams, §3.7) but not stated as an explicit 'intended users' section

No description of a systematic literature search strategy

No explicit evidence selection criteria described

Quantitative appraisal is shown for the core RCT evidence (Cochrane meta-analysis results with RR, 95% CI, NNT reported in §3.1), but this is not extended systematically to all recommendations

No description of how recommendations were formulated (no GRADE, consensus/Delphi method, or voting described)

Benefits/risks explicitly weighed: dedicated side-effect section, contraindications, drug-interaction table, and system-by-system risk management (Table 3)

Strong in-text numbered citation linking specific statements to 27 references, including exact effect estimates from the cited Cochrane review

No explicit statement of external peer review; the 7-person editorial board alongside 3 authors suggests internal review, but this is not stated as an external process

General intent to update 'in future years as evidence develops' is stated on the copyright page, but no specific procedure or interval is given

Mostly specific and quantitative, though the guideline itself acknowledges a genuine grey zone (Section 4) for cases not meeting standard criteria, which is transparent but reduces unambiguity

Options clearly contrasted: active vs passive cooling, whole-body vs selective head cooling (with a pros/cons discussion), stepwise anticonvulsant escalation

A dedicated numbered 'Sonuç ve Öneriler' (Conclusions and Recommendations) section distills the 10 key take-home recommendations

Facilitators/barriers addressed via coordination with regional emergency transport systems and referral pathways between centres without cooling devices (Recommendations 1 and 6)

Very strong implementation toolkit: 3 flowcharts (passive cooling, seizure management, summary algorithm), drug-dosing/pharmacokinetics table, and a model informed-consent form

Only a passing remark that servo-controlled systems 'may be somewhat more expensive' (§3.10); no systematic cost or resource-implication analysis

Follow-up is recommended (Amiel-Tison at discharge; Bayley II/III long-term) and centres are told to keep follow-up/referral documentation, but no structured audit schedule is given

No funding source or statement on funder influence is reported anywhere in the document

No competing-interest disclosure or management process is reported for the authors/editors

**Rater 2:** When inclusion criteria are unclear, recommendations inevitably become ambiguous.

A variety of options was presented; however, no clear evidence was provided, and there was a lack of clarity regarding when to adopt alternative treatments.

**SLOVAKIA**

***Free comments***

**Rater 1:** Objective stated in a single explicit sentence (reduce mortality, improve long-term prognosis in HIE II-III) but not elaborated further

Health question well covered via a structured decision algorithm (inclusion criteria A/B/C, contraindications) even though not phrased as a formal PICO

Population very precisely specified: GA ≥35 wks, BW ≥1800g, <6h onset, HIE severity criteria, explicit contraindications

No information on the composition of the development group is reported

No evidence that views/preferences of patients or families were sought during development

Target users implied (transport/NICU teams of the 7 named cooling centres) but not explicitly stated

No systematic literature search methodology described

No evidence selection criteria described

No appraisal of strengths/limitations of the evidence base

No description of how recommendations were formulated (no consensus method, GRADE, etc.)

Risk/benefit balance is implicitly addressed via the contraindications and complications sections, but not through a formal evidence-based weighing

A 14-item reference list is provided, but recommendations are not individually linked to supporting citations

No mention of external review prior to publication

No updating procedure specified

Recommendations are highly specific and unambiguous, with exact temperatures, timings and thresholds throughout

Alternative management options are clearly presented (servo-control vs SW-control cooling, cooling with vs without a device, two warming methods)

Well structured with headers/algorithm/tables, but no distilled 'key recommendations' summary; density makes rapid orientation harder

Facilitators/barriers to applying the protocol are not discussed

Extensive practical tools provided: stepwise procedures, probe-insertion technique, scoring appendices, decision algorithm

Equipment lists are given but costs/resource implications are not discussed

Detailed monitoring/audit schedule with a day-by-day laboratory table through day 10

No funding source or statement on funder influence is reported

No competing-interest disclosure or management process is reported

**NORWAY**

***Free comments***

**Rater 3:** The guideline acknowledges several areas where evidence is limited, including treatment of mild encephalopathy, infants outside standard eligibility criteria, and adjuvant neuroprotective therapies. However, it does not provide a structured discussion of the limitations

the guideline provides several supporting clinical resources, including algorithms, tables, practical management tools, an informed-consent form, and a reference list. However, no methodological report, evidence profiles, evidence tables, or supplementary development documents are made available

**Rater 4:** I can only see their names, not their professions

although the guideline states that it is updated at 2022, there is some references to a paper published in 2025
